# Ethnic Related Selection for an *ADH* Class I Variant within East Asia

**DOI:** 10.1371/journal.pone.0001881

**Published:** 2008-04-02

**Authors:** Hui Li, Sheng Gu, Xiaoyun Cai, William C. Speed, Andrew J. Pakstis, Efim I. Golub, Judith R. Kidd, Kenneth K. Kidd

**Affiliations:** 1 Lab for Human Polymorphism Studies, Department of Genetics, School of Medicine, Yale University, New Haven, Connecticut, United States of America; 2 MOE Key Laboratory of Contemporary Anthropology and Center for Evolutionary Biology, School of Life Sciences and Institutes of Biomedical Sciences, Fudan University, Shanghai, China; University of Utah, United States of America

## Abstract

**Background:**

The alcohol dehydrogenases (ADH) are widely studied enzymes and the evolution of the mammalian gene cluster encoding these enzymes is also well studied. Previous studies have shown that the ADH1B*47His allele at one of the seven genes in humans is associated with a decrease in the risk of alcoholism and the core molecular region with this allele has been selected for in some East Asian populations. As the frequency of ADH1B*47His is highest in East Asia, and very low in most of the rest of the world, we have undertaken more detailed investigation in this geographic region.

**Methodology/Principal Findings:**

Here we report new data on 30 SNPs in the *ADH7* and Class I *ADH* region in samples of 24 populations from China and Laos. These populations cover a wide geographic region and diverse ethnicities. Combined with our previously published East Asian data for these SNPs in 8 populations, we have typed populations from all of the 6 major linguistic phyla (Altaic including Korean-Japanese and inland Altaic, Sino-Tibetan, Hmong-Mien, Austro-Asiatic, Daic, and Austronesian). The *ADH1B* genotyping data are strongly related to ethnicity. Only some eastern ethnic phyla or subphyla (Korean-Japanese, Han Chinese, Hmong-Mien, Daic, and Austronesian) have a high frequency of ADH1B*47His. *ADH1B* haplotype data clustered the populations into linguistic subphyla, and divided the subphyla into eastern and western parts. In the Hmong-Mien and Altaic populations, the extended haplotype homozygosity (EHH) and relative EHH (REHH) tests for the *ADH1B* core were consistent with selection for the haplotype with derived SNP alleles. In the other ethnic phyla, the core showed only a weak signal of selection at best.

**Conclusions/Significance:**

The selection distribution is more significantly correlated with the frequency of the derived *ADH1B* regulatory region polymorphism than the derived amino-acid altering allele ADH1B*47His. Thus, the real focus of selection may be the regulatory region. The obvious ethnicity-related distributions of *ADH1B* diversities suggest the existence of some culture-related selective forces that have acted on the *ADH1B* region.

## Introduction

Historically, the alcohol dehydrogenases (ADH) have been among the most widely studied sets of enzymes along with their genes. Alcoholism, a complex genetic disorder that affects a large proportion of people, has been known for some time to be strongly associated with variants of alcohol dehydrogenase [1]–[3]. Alcohol dehydrogenase plays a role not only in alcohol metabolism but also in many other metabolic pathways, and thus forms of the enzyme exist in many organs [4]–[8]. The human *ADH* gene cluster is located on chromosome 4q23-24, and the several genes are clearly related evolutionarily. Sequentially this *ADH* cluster contains *ADH7, Class I ADH (1C, 1B, 1A), ADH6, ADH4, ADH5* [9], [10]. Hundreds of polymorphic sites have already been studied within the *ADH* cluster [11], [12]. Some polymorphic sites alter amino acids or lie in other functional regions; these can cause functional changes in the enzyme and result in different phenotypes [11], [13]–[20]. The Arg47His (rs1229984) polymorphism at *ADH1B* (previously *ADH2*) is a typical function-related polymorphism [21], [22]. The derived allele, coding for histidine, provides a well confirmed protection against alcoholism [23]–[29].

Global investigation of *ADH1B* diversity shows a strong geographic distribution [10], [30]. Some variants of *ADH1B* appear specifically in some geographic regions [10], [24], [31]–[33]. For instance, ADH1B*47His reaches very high frequencies almost exclusively in East Asian populations [10], [12], [34] while fairly high frequencies occur in West Asia and North Africa; in contrast the allele is rare to absent in the rest of the world [30]. This quite unusual geographic pattern argues for more detailed research. Linkage disequilibrium (LD) studies revealed evidence that the upstream region of *ADH1B* has been under positive selection in several East Asian populations such as Chinese, Koreans and Japanese, but the selective force remains unknown [12]. There are still unsolved issues in relating the high frequency of the functional variant to positive selection in East Asian populations. For example, in some Austronesian (AU) populations, the derived allele frequency of the functional polymorphism is very high, but there is no evidence by the REHH test showing that the functional allele ADH1B*47His underwent selection [12]. Thus, not only is the selective force unknown, but selection cannot explain the frequency of ADH1B*47His in all populations in which it is high. While absence of evidence in some populations may have been a power issue, further investigation is clearly needed.

Our previously studied populations belong to several ethnic phyla (same as linguistic phyla): the Korean-Japanese (KJ) subphylum of the Altaic phylum (Japanese, Koreans), the Chinese (also called Sinitic Han: SN) subphylum of the Sino-Tibetan phylum (Cantonese, Hakka and Minnam Taiwanese), the Taiwan subphylum of the AU phylum (Atayal, Amis), and the East Mon-Khmer subphylum of the Austro-Asiatic (AA) phylum (a small sample of Cambodians) [12]. These eight populations are all located in the coastal region. According to the complicated landforms, climates, ethnic distributions, and population histories in East Asia, this set of population samples is not sufficiently representative of East Asia, especially not for the populations in the western region of East Asia such as the Tibetan, Uigur, Mongol, etc., populations. Based on the geographic distribution of the ADH1B*47His allele [30], we do not believe that selection happened everywhere in East Asia, certainly not to the same effect. Therefore, we are working to improve coverage of other East Asian ethnic groups and geographic regions to reveal the true histories of ADH genes [30]. More detailed distributions of allele and haplotype frequencies will be essential for attempting to address the questions of when, where, and even how selection occurred, although the distributions alone will not allow definitive answers.

The cultural and ethnic diversity in East Asia is noticeable. For example, Daic (also called Tai-Kadai: TK) is the major phylum in the peninsula of Southeast Asia and southern East Asia. In northern East Asia, Altaic is the major phylum, but the KJ subphylum is an atypical branch. Inland Altaic (AT) subphyla such as Mongolian and Turkic are much more representative of Altaic. In the western side of East Asia, the Tibeto-Burman (TB) subphylum and the Hmong-Mien (HM) phylum are both dominant [35], [36]. Different ethnic phyla not only have different languages and religions but also completely different life styles [36], [37]. Almost all of those AT populations are nomandic tribes except Uigur, which switched to farming 1000 years ago [38], [39]. The northern tribes of TB are all mainly pastoralists. TK, HM, SN, KJ, and AU populations have very long histories of farming [40]–[42]. Most of the AA (mainly Mon-Khmer) groups were hunter-gatherers historically, and began farming very recently [43]. Different life styles might have led to differences in the impacts of any selective forces, and might have influenced the distribution of *ADH* allele and haplotype frequencies.

**Figure 1:**
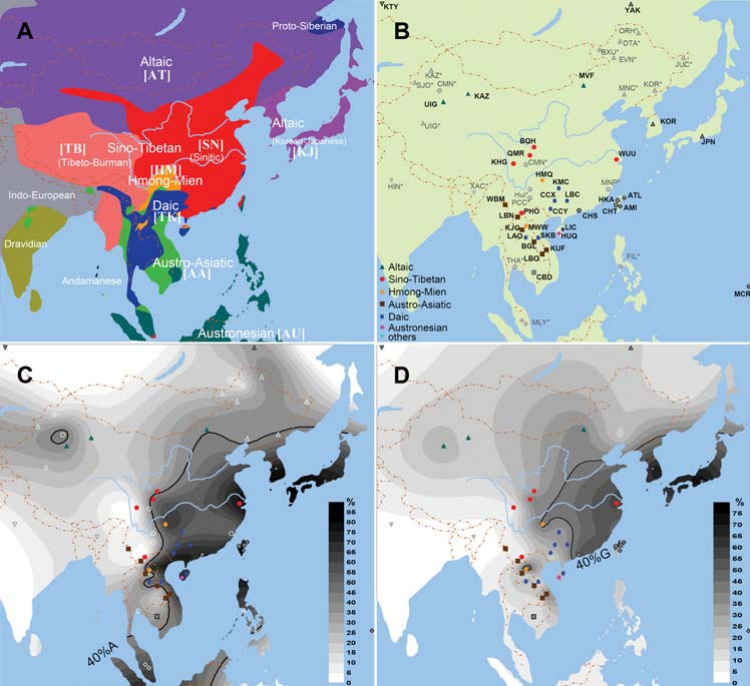
Locations of the populations and distributions of the *ADH1B* rs1229984 and rs3811801 derived allele frequencies. Note: The map of part A showed the ethnic phyla in East Asia, and part B displayed the locations of the populations. Populations marked with stars were cited from literature [31], [34]. The codes of the star-marked populations are ISO639-3 codes. Populations shown by gray spots are previously published by our team [12]. The colorful spots are the populations collected in this study. Part C is the distribution of the derived allele frequency of rs1229984 (ADH1B*47His). Part D is the distribution of the regulatory region polymorphism rs3811801 derived allele frequency.

To determine the allele frequency distributions with greater geographic and ethnic precision, we have studied 24 more populations from different geographic areas of East Asia. These populations belong to different ethnic phyla and their major subphyla. The most important populations of TK, AT, HM, TB, and AA phyla are included (Figure 1A, 1B and Table 1). This type of sampling can minimize the bias of the geographic and ethnic distribution of the samples and the possible misreading of the results. With such data we may be able to identify selection on the relevant allele and to find relationships between haplotype pattern and selection, as much more genetic diversity may be found during this more comprehensive investigation in the focused East Asia region.

**Table 1:** General Information for the East Asian populations included in the analyses

Ethnic Phylum	Subphylum	Code	Population name	sample size	Country	Province	County	Long.(E)	Lat.(N)
Altaic	Turkic	KAZ*	Khazaks	48	China	Xinjiang	Balikun	93.01	43.59
Altaic	Turkic	YAK	Yakut	51	Russia	Saha		124.20	62.07
Altaic	Turkic	UIG*	Uigur	48	China	Xinjiang	Turpan	88.66	42.79
Altaic	Mongolian	MVF*	Mongols	75	China	Inner Mongol	Shilingol	116.07	43.95
Altaic	Korean-Japanese	KOR	Koreans	54	S.Korea			126.57	37.32
Altaic	Korean-Japanese	JPN	Japanese	47	Japan			139.49	35.38
Sino-Tibetan	Tibeto-Burman	BQH*	BaimaDee	42	China	Sichuan	Pingwu	104.53	32.41
Sino-Tibetan	Tibeto-Burman	QMR*	Qiang	40	China	Sichuan	Mao	103.85	31.69
Sino-Tibetan	Tibeto-Burman	KHG*	Khamba Tibetan	36	China	Sichuan	Kangding	101.96	30.05
Sino-Tibetan	Tibeto-Burman	PHO*	Phunoi	43	Laos	Louang-Namtha	Louang-Namtha	101.05	21.13
Sino-Tibetan	Sinitic(Han Chinese)	WUU*	Wu Chinese	53	China	Shanghai		121.37	31.11
Sino-Tibetan	Sinitic	HKA	Hakka Chinese	41	China	Taiwan		121.05	24.20
Sino-Tibetan	Sinitic	CHT	Minnam Chinese	50	China	Taiwan		120.31	23.31
Sino-Tibetan	Sinitic	CHS	Canton Chinese	57	USA	CA	San Francisco	113.04	22.35
Hmong-Mien	Hmongic	HMQ*	Black Hmong	60	China	Guizhou	Mashan	109.80	27.88
Hmong-Mien	Hmongic	MWW*	White Hmong	60	Laos	Huapuan	XamTai	103.54	19.57
Daic	Kam-Sui	KMC*	Kam	74	China	Guangxi	Sanjiang	109.60	25.79
Daic	Kam-Sui	LBC*	Laka	98	China	Guangxi	Jinxiu	110.18	24.13
Daic	Tai-Sek	CCX*	North Zhuang	40	China	Guangxi	Wuming	108.28	23.17
Daic	Tai-Sek	CCY*	South Zhuang	30	China	Guangxi	Chongzuo	107.36	22.42
Daic	Hlai	LIC*	Hlai	59	China	Hainan	Tongzha	109.52	18.77
Daic	Tai-Sek	LAO*	Lao	117	Laos	Cap.Vientiane	Sisattanak	102.37	17.57
Daic	Tai-Sek	SKB*	Saek	57	Laos	Khammouan	Boualapha	104.55	17.28
Austro-Asiatic	North Mon-Khmer	WBM*	Ava	59	China	Yunnan	Ximeng	99.46	22.74
Austro-Asiatic	North Mon-Khmer	KJG*	Khmu	51	Laos	LouangPrabang	Nambak	102.33	20.27
Austro-Asiatic	North Mon-Khmer	LBN*	Lamet	42	Laos	Louang-Namtha	Nale	101.35	20.50
Austro-Asiatic	Viet-Muong	BGL*	Bo	52	Laos	Bolikhamxai	Khamkheut	105.09	18.08
Austro-Asiatic	East Mon-Khmer	KUF*	Katu	50	Laos	Xekong	Thateng	106.39	15.36
Austro-Asiatic	East Mon-Khmer	LBO*	Laven	47	Laos	Xekong	Thateng	106.35	15.34
Austro-Asiatic	East Mon-Khmer	CBD	Cambodians	25	Cambodia			104.55	11.33
Austronesian	Malayo-Polynesian	HUQ*	Tsat	52	China	Hainan	Sanya	109.27	18.17
Austronesian	Atayalic	ATL	Atayal	42	China	Taiwan	Yilan	121.74	24.76
Austronesian	Paiwanic	AMI	Amis	40	China	Taiwan	Hualien	121.60	23.98
Austronesian	Malayo-Polynesian	MCR	Micronesians	37	Micronesia			158.12	6.57

Note: ^*^Those populations marked with stars are newly collected, and the triliteral codes are ISO639-3 codes [35]. Others are previously published populations with previously used codes [12]. San Francisco Chinese originally came from Jiangmen County, Guangdong, China; therefore, the location of CHS in the table is that of Jiangmen County.

## Results

### Distribution of ADH1B variants

**Figure 2:**
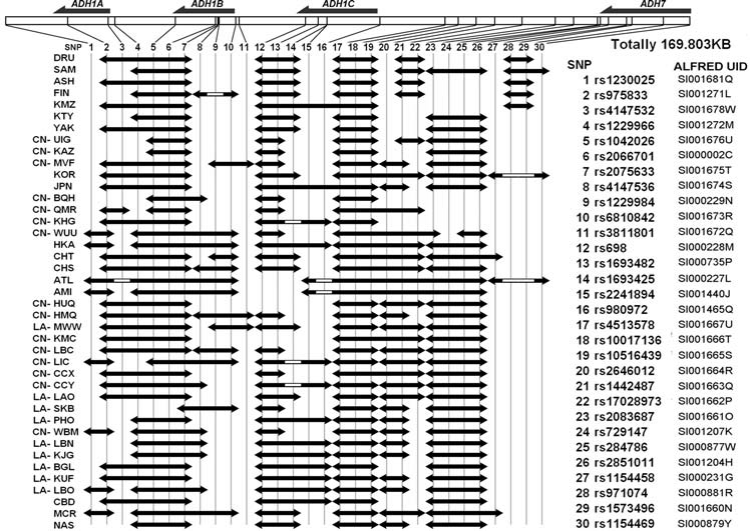
Pattern of regions of high LD using HAPLOT and the default r^2^ algorithm. Note: The codes of the non-East Asian populations are shown as NAS(Nasioi), KTY(Khanty), KMZ(Komi), FIN(Finns), SAM(Samaritans), DRU(Druze), ASH(Ashkenazi Jews). CN: New collected samples from China. LA: Newly collected samples from Laos. Both dbSNP numbers and ALFRED UID numbers are presented for the SNPs in the ADH region we typed.

We obtained the allele frequencies of 30 single nucleotide polymorphisms (SNPs) on the 24 new populations. The haplotype frequencies for all the 30 SNPs are given in Table S1 and allele frequencies are in ALFRED under the UIDs in Figure 2. The frequencies of the *ADH1B* functional variant, ADH1B*47His, and the variant at the *ADH1B* regulatory polymorphism rs3811801 (SNPs 9 and 11) were transformed into contour maps in Figure 1. The sharp borders across which the frequencies changed quickly are marked by bold lines in the map. The distributions of both variants show very clear clinal geographic patterns of east-west division. The sharp border for ADH1B*47His lies between the eastern part and western part of both the continental East and Southeast Asia, from the south end in Cambodia to the north end in Inner Mongol. The frequencies of ADH1B*47His are quite high to the east of this border. The rs3811801 derived allele shows a somewhat different distribution within the range of ADH1B*47His with a less sharp border and a similar higher frequency to the east.

Ethnically, the distributions of these two variants are also quite regular. The populations with high frequency of ADH1B*47His belong to TK, AU, SN, KJ, and HM. Those subphyla all have a long history of agriculture [40]–[42], while those populations with a low frequency of ADH1B*47His are all pastoral or hunting populations or began to farm recently. The TK and AU populations are excluded from among the populations with a high frequency of the rs3811801 (SNP 11) derived allele.

This non-synonymous SNP and the *ADH1B* promoter [44] polymorphism (SNP 11) have very high F_st_ values on a global scale [12]. The fixation index F_st_, originally designed as the most inclusive measure of population substructure, is used here as a measure of allelic difference among the populations [10]. As the populations in East Asia are fairly similar to each other [45]–[47], the F_st_ values within the region are expected to be lower than those of the same SNPs globally. Using data on the East Asian populations listed in Table 1, we calculated the F_st_ values of all the SNPs as follows, numbered as in Figure 2: 1(.056), 2(.196), 3(.185), 4(.153), 5(.182), 6(.177), 7(.182), 8(.051), **9(.240)**, 10(.098), **11(.218)**, 12(.145), 13(.145), 14(.198), 15(.236), 16(.145), 17(.166), 18(.166), 19(.166), 20(.094), 21(.203), 22(.168), 23(.084), 24(.101), 25(.089), 26(.101), 27(.057), 28(.053), 29(.154), 30(.225). The F_st_ values among East Asian populations are much lower than the global values of the same SNPs, though the F_st_ of our focused SNPs 9 and 11 are still much higher than the global F_st_ mean of general SNPs (0.14). The F_st_ values of the Arg47His variant and the promoter variant of *ADH1B* are among the highest values, but we also see high values in the upstream region at *ADH1C* and even upstream of *ADH1C*.

### Haplotypes and regions of high LD

**Figure 3:**
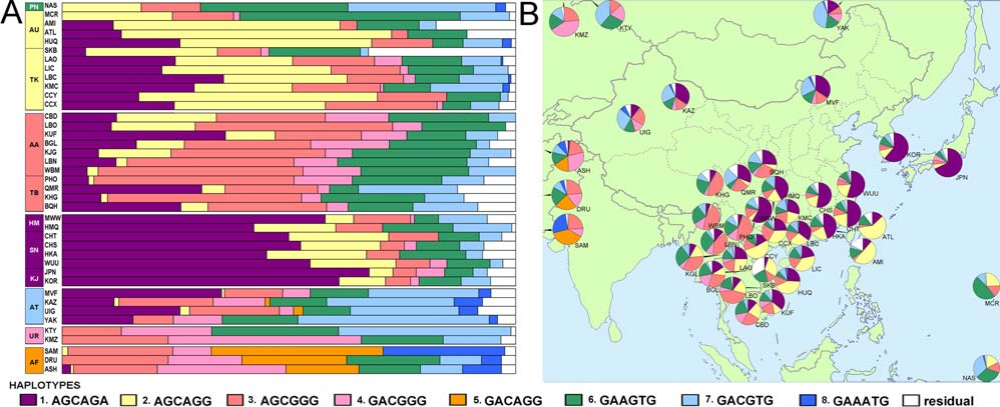
Haplotype frequencies of the *ADH1B* gene region including the regulatory region. Note: the SNPs in the haplotypes are rs2066701-rs2075633-rs4147536-rs1229984-6810842-rs3811801, corresponding to SNPs 6–11 in Figure 2. Phyla, PN: Papuan-New Guinean, AU: Austronesian, TK: Daic, AA: Austro-Asiatic, TB: Tibeto-Burman, HM: Hmong-Mien, SN: Sinitic Han, KJ: Korean-Japanese, AT: Altaic (inland), UR: Uralic, AF: Afro-Asiatic. The patterns of the non-East Asian phyla (UR, AF, PN) are quite different from those of East Asian phyla. The patterns of the phyla in East Asia can be classified into four groups as the colors shown in the left side bar. The frequency data for all haplotypes are in Table S1.

Six-SNP haplotypes of the *ADH1B* upstream region (SNPs 6–11) were estimated and the haplotype frequencies of the Asian populations are displayed in Figure 3. The patterns of the East Asian populations are obviously different from the non-East Asian populations (Uralic and Afro-Asiatic). Haplotypes 1 and 2 have high frequencies in most of the East Asian populations, while they occurred at much lower frequency in ASH (Jews). The populations in the most southwest region of East Asia (WBM: Ava, KHG: Tibetan, and PHO: Phunoi Lolo) have the lowest frequencies of these two haplotypes.

Among the East Asian populations, there are four types of haplotype patterns, and the classification shows an obvious ethnic correlation. The first group (Southeast) contains AU and TK, and haplotype 2 is the major haplotype of this group. The second group (Southwest) contains AA and TB, with the characteristic haplotype 3. Haplotype 1 has a frequency greater than 50% in the third group (Northeast) that contains HM, SN, and KJ. AT populations form the fourth group (Northwest). The haplotypes in group 4 are most diverse. Only haplotype 7 is a little richer than it is in the other groups. For those non-East Asian populations, the patterns also seem regular. Haplotypes 5 and 8 are frequent in Afro-Asiatic populations but absent in Uralic populations.

Regions of high LD across the whole region were displayed in Figure 2. The patterns of the *ADH7* region and the region between *Class I ADH* and *ADH7* (SNPs 17–30) are quite similar among the populations in East Asia, except for TB (BQH, QMR, KHG) with fewer regions of high LD and AU (ATL, AMI) with larger high LD regions. In contrast, the LD patterns of *Class I ADH* (SNPs 1–16) are quite different among the populations. It is interesting that only the Mongol (MVF) and Hmong (HMQ, MWW) have high LD extending to the region upstream of *ADH1B* (SNPs 9–11). In the two AU populations from Taiwan, high levels of LD encompass more SNPs than seen for the other populations; this pattern suggests they are quite young and/or have undergone considerable random genetic drift recently.

### Population Comparison

**Figure 4:**
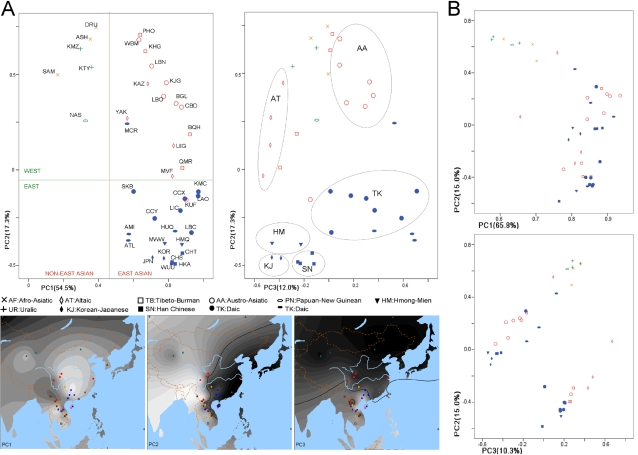
Principal Component Analysis plots. Notes: The plots show the relationships among populations estimated by PCA. Plots in part A were based on the *ADH1B* haplotypes frequency data in Figure 3. Plots in part B used the haplotype frequency data of the whole *ADH* region in Figure 2. In part A, an ethnic related distribution is obvious, while in part B the distribution shows no strong distinct clusters corresponding to ethnicity.

A principal component analysis based on the *ADH1B* haplotype frequencies in Figure 3 was used to examine the data distribution among populations (Figure 4A). The first plot was constructed by principal components (PC) 1 and 2. In this plot, PC1 divides East Asian populations from non-East Asian populations. This confirms the overall genetic unity and distinctiveness of East Asia. In the second map PC2 divides the western part of East Asia from the eastern part with a sharp border. The distribution of PC2 is very similar to the distribution of ADH1B*47His, with the TK, HM, SN and KJ phyla in the east and the others in the west. The correlation between PC2 value and ADH1B*47His frequency is −0.971 (P<0.001), while the correlation between PC2 and longitude is −0.491 (P = 0.005). The distribution of PC3 is also quite geography-related. It divides southern phyla (TK, AA, AU) from the northern phyla. The correlation coefficient between PC3 and the latitude is −0.808 (P<0.001). Though PC2 and PC3 are significantly correlated with geography, they are more ethnic-related, as is shown in the second plot of Figure 4A. It is noticeable that those eastern populations such as LAO and MWW that have moved into the western area are still clustered with the eastern phyla. That suggests the distribution of this *ADH1B* haplotype (encompassing the 5′ half of the gene and the 5′ flanking region) must be related to the history of the ethnic phyla.

To test if the ethnic-related distribution of *ADH1B* is common in the human genome, we applied principal component analysis (PCA) to the haplotype frequency data of the whole *ADH* region we typed. Results are shown in Figure 4B. The distribution of the PCs is much closer to the general genetic relationship (a supposed genetic relationship measured by the whole genome diversity) among populations than the *ADH1B* distribution. PC1 still divides East Asia from the rest of the world. Neither PC2 nor PC3 is related to ethnicities. The PCA result of the whole region is very different from that of the smaller *ADH1B* region. Therefore the ethnic-related distribution of the first PCA of ADH1B is uncommon. The distribution of the *ADH1B* upstream region diversity is related to ethnicity, but different from the distribution of the whole Class I *ADH* and *ADH7* region diversity.

### Positive Selection Test of ADH1B Core Haplotypes

**Figure 5:**
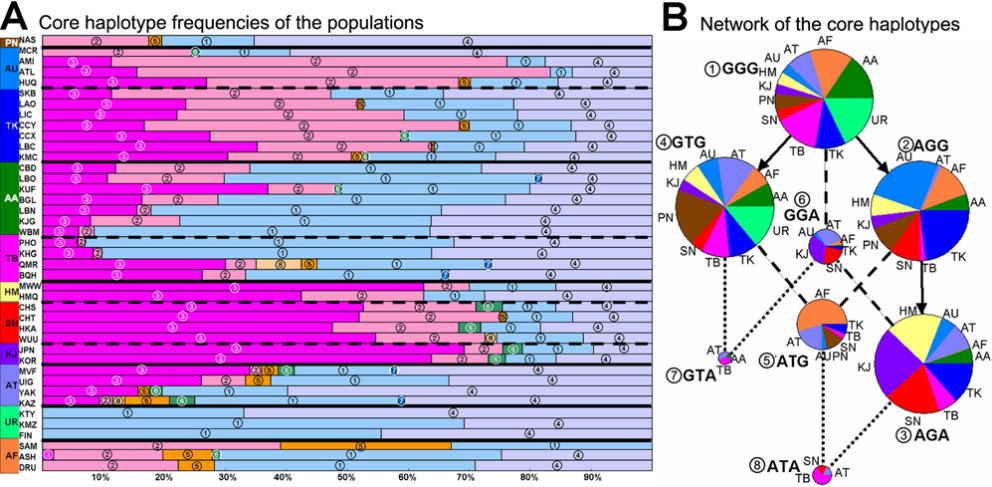
Frequencies and network of the core haplotypes (rs1229984-rs6810842-rs3811801). Notes: Haplotype codes (1) to (8) are in the same system for both part A and part B of the figure. The sizes of the balls in the network of part B represent the rough relative frequencies of the haplotypes. The arrows are the most likely mutational relationships. The broken lines indicate possible historical recombinations. Haplotype (3) is more derived and presumably younger than haplotypes (1), (2), (4), and (6). Its high frequency in some populations suggests selection may have operated.

As SNPs 9 to 11 are candidates for being related to function or having been positively selected, we chose this short region as the core for a test for selection. The allele frequencies of the core haplotypes are shown graphically in Figure 5A. The four-group classification of Figure 2 can still be observed in Figure 5A. There are eight haplotypes but only four are common in East Asia. Haplotype (2) is most common in the AU-TK group. Haplotype (3) reaches highest frequency in the HM-SN-KJ group. Uralic and Afro-Asiatic are obviously different. Though AT and Uralic phyla are neighbors and share much of their culture and customs, their haplotype patterns are quite different.

In Figure 5B, the relationships among the haplotypes are plotted in a network. Haplotype (1) is the ancestral haplotype based on sequence comparison with four other ape species. The functional mutation ADH1B*47His occurred in the step from (1) to (2). The promoter mutation occurred in the step from (2) to (3). The sizes of the circles in Figure 5B are roughly proportional to the haplotype frequencies in the different ethnic phyla. In the network, the frequencies of the younger haplotypes are expected to be lower. Actually, young haplotypes (5) to (8) are obviously less frequent than those old ones (1), (2) and (4). The only exception is haplotype (3) which is young but still reaches very high frequency in East Asia. Outside of East Asia, this haplotype is very rare. This indicates this haplotype has undergone strong genetic drift or positive selection in East Asia. This also focuses attention on the derived allele in the promoter region.

**Figure 6:**
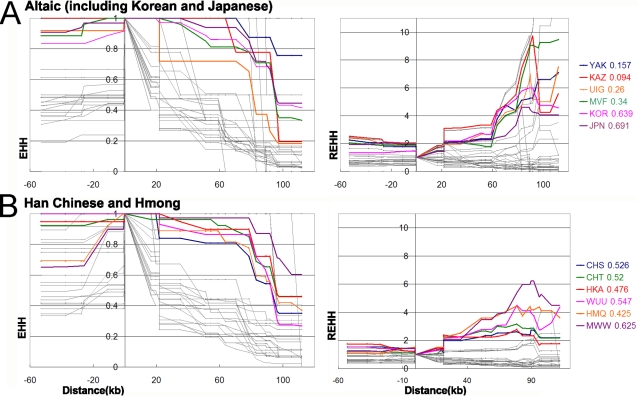
Extended Haplotype Homozygosity (EHH) and Relative Extended Haplotype Homozygosity(REHH) of Altaic, Han and Hmong populations. Note: Colorful lines are data of core haplotype (3)AGA, and gray lines are data of other haplotypes. The data following the population codes are frequencies of the core haplotype in the populations. EHH and REHH of the other populations are in Figure S1.

To test whether the high frequency of the young haplotype was caused by positive selection, we applied the EHH and REHH tests. The results are presented in Figure 6 and Figure S1 to scale along the chromosomal segment studied. If the EHH value decayed quickly or the REHH value did not rise to high levels with increasing distance from the core, there would be no evidence for selection. The EHH values of core haplotype (3), AGA, decayed more slowly than the other core haplotypes, indicating haplotype (3) may have been under positive selection. The REHH values of haplotype (3) also increased in the upstream direction. The REHH values of the Altaic populations including KJ were the highest among the ethnic phyla. The REHH values of two HM populations, BQH, CBD and SKB also rose to fairly high values. The high REHH values suggest the possible existence of positive selection; however, because of limitations of our sample sizes (∼50 individuals per population) only those core haplotypes of moderate frequency convey meaningful results. Some populations with high REHH values failed to have moderate haplotype frequencies, such as YAK, KAZ, CBD and SKB. Thus, we cannot draw a definite conclusion for these populations.

**Figure 7:**
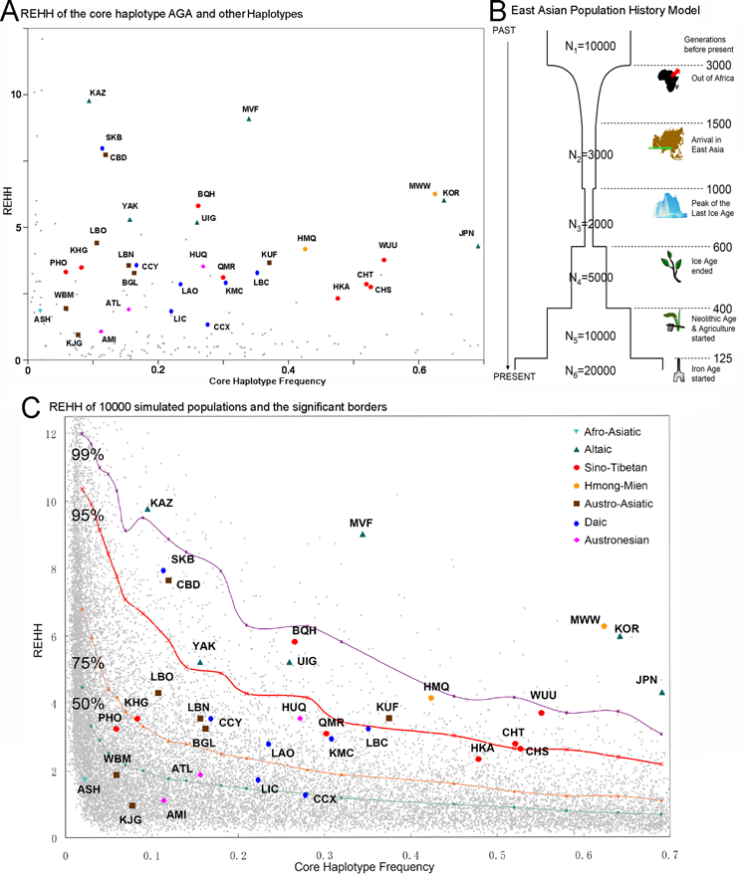
REHH of observed and simulated populations. Note: The colored dots are observed REHH data of core haplotype AGA both in chart A and C. In chart A, the observed REHH data shows that most of the REHH values of haplotype AGA are higher than those of the other haplotypes. Part B is the East Asian population history model determined by complicated factors. Six phases were defined with the effective population numbers and the generation numbers to present. Chart C indicates the REHH data simulated by the model in part B along with the data in Chart A. The lines in chart C are comparison borders of the simulated data. The observed REHH of haplotype AGA of all the Altaic and Hmong populations are above the 95% border, which is the evidence of positive selection.

**Figure 8:**
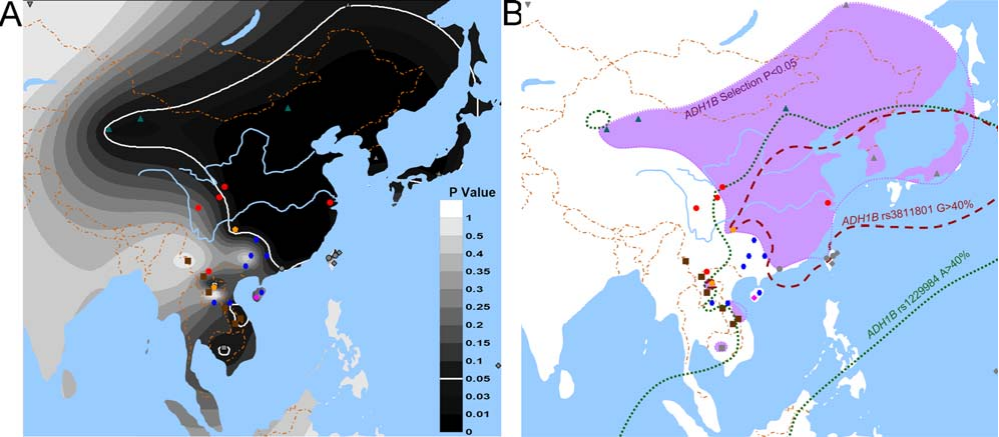
Significance P values of the positive selection on the *ADH1B* gene in East Asia. Note: The map of part A displayed the distribution of the significance P value of the positive selection on the *ADH1B* gene. Populations from most areas of East Asia have been significantly selected for except those in the southwest. In part B, the selection area was compared with the high frequency areas of two *ADH1B* SNPs. The dotted line encloses the region in which the ADH1B*47His frequency is >40%, and the broken line encloses the region in which the *ADH1B rs3811801* frequency is >40%. The distributions of ADH1B*47His and selection area differ from each other, which indicates that they are unrelated. The high frequency area of rs3811801 derived allele is included in the selection area, indicating the possible effect of this allele in the selection on the *ADH1B* gene.

We plotted all the REHH values for all core haplotypes at SNP 26 (around 90 kb from the core) in Figure 7A. In Altaic and some other populations, the REHH values of haplotype (3), AGA, were much higher than those of the other haplotypes. To test whether this haplotype might have been under positive selection in these populations, we simulated the population demography (Figure 7B). The population history model used in the simulation was based on the migration history reconstructed by other genetic studies (mtDNA [46] and Y-chromosome [47]) and the tremendous changes of the environment or the society in East Asia. The population size was at its lowest level at the peak of the ice age [48], and increased quickly when agriculture started. Archaeological evidence [41] confirms that agriculture started more than 8000 years ago in East Asia. If we assume a generation is about 20 years, agriculture started 400 generations ago. With this model, 10,000 populations were simulated, and the results of simulated REHH values were shown in Figure 7C together with the observed REHH values of haplotype AGA. Four lines (50, 75, 95 and 99 percentile) were drawn for visual comparison. All of the Altaic and HM populations are above the 95 percentile line. We also calculated the significance by a *t*-test after transforming our original result to achieve normality. The P values were transformed into the contour map in Figure 8A. There are 15 populations with a P value less than 0.05. The regions covered by these populations are marked in Figure 8B by a purple background. All of the Altaic populations and HM populations are included. Among four SN populations only HKA (Hakka) is excluded. BQH, SKB, CBD and KUF are also included. The sample size of CBD is rather small (25), and the core haplotype AGA frequency is low; therefore the REHH of CBD is not reliable. Fisher's Exact Test [49] on haplotype composition shows CBD can be combined with LBO, a nearby and similar population (P = 0.492). REHH of the combined population is 4.467, which suggests that the combined population shows no evidence of selection. Therefore the populations with significant evidence of selection are ethnic-specific with only four exceptions. The conclusion is that strong evidence of selection on the core haplotype AGA was found in Altaic and HM populations, and weak evidence of selection in SN.

In the map of Figure 8, we can see that the geographic area with evidence of selection is quite different from the ADH1B*47His high frequency area. Correlation analyses were computed between the P values of the selection significance test and the derived allele frequencies of the SNPs in the core region. The results were SNP 9 (r = 0.100, P = 0.581), SNP 10 (r = 0.097, P = 0.590), **SNP 11 (r = 0.522, P = 0.002)**. Only SNP 11 has significant P values for selection. SNP 11 (rs3811801) is located at −1761 bp, the promoter region of *ADH1B* [44]. Therefore the promoter region may be the actual region that has undergone selection. The alleles at this SNP may quantitatively alter *ADH1B* expression. However, since the derived allele at SNP 11 (rs3811801) occurs only on chromosomes with the ADH1B*47His allele, the cis-acting combination of the two alleles may have been the focus of selection.

## Discussion

### Population History and Ethnic-related Distribution of ADH1B

In all of the analyses based on the *ADH1B* region in this study, we saw ethnic relationships with allele and haplotype frequencies. Especially in Figure 4A of the PCA analysis, genetic clustering of populations matched very well with the ethnic phyla or subphyla. That is rather unusual, as we rarely see the distribution of alleles at a single autosomal locus matching finer structure of ethnic classification. There are many papers describing ethnic differences for certain loci, but those ethnic differences are among populations from different geographic regions of the world [50]–[52]. In our study on *ADH1B*, the eight ethnic phyla or subphyla included are all in East Asia. In the PCA, these eight groups can be distinguished very well by *ADH1B* data, but not by the whole Class I *ADH* and *ADH7* region data. The ethnic-related patterns are only seen for the *ADH1B* region.

In East Asia, one hypothesis argues that most of the homologous populations in one ethnic phylum share a distinct common ancestor, which has some evidence from Y chromosome DNA [53]–[55]. As the paternal societies have lasted for a very long time in most of the East Asian populations, the Y chromosome had less chance to flow among populations than other chromosomes. Therefore the distribution of Y chromosome diversity can represent the original relationships among the ethnic phyla to a certain extent. On the other hand, two factors can change the ethnic-related genetic structure. Interactions among different ethnic phyla in East Asia have frequently occurred. Historical facts of interactions were often used to explain the different genetic structures revealed by autosomal [56] and mitochondrial [46] DNA diversity. At the same time, random genetic drift will change the frequency of a single locus in a certain population. Therefore most of the distributions of any single locus are not as ethnically related as the Y chromosome is. The ethnically related distribution of *ADH1B* cannot be explained only by random genetic drift. There can be two possible explanations. One is that some culture related incidence has maintained the special distribution. Another possible explanation is that different *ADH1B* haplotype patterns occurred at the founding of the different ethnic phyla and remained largely unchanged during population expansions and interactions.

A number of Y chromosome diversity studies showed that TB, SN, TK and AU phyla could be recognized by some ethnicity-specific haplogroups [53], [54], [57]. TB and SN subphyla belong to Sino-Tibetan phyla, and they both originated from the upper Yellow River area and shared a common ancestor [53]. TK and AU are two different ethnic phyla, but a great deal of evidence showed that they are very similar to each other [58]. Y-chromosome data also supports a common origin of TK and AU [57]. In our *ADH1B* data, TB and SN departed from each other, while TK and AU clustered closely. *ADH1B* data do not always match the hypothesized original relationships. Therefore the ethnic-related structure of *ADH1B* might not always be caused by the original differentiations of the ethnic phyla. Some cultural factors such as life styles may have influenced the special distribution of *ADH1B*, especially in the Sino-Tibetan phylum. Weak evidence of selection was seen in the SN subphylum. That implies the *ADH1B* haplotype pattern in SN may be due to selection, and the selective forces on *ADH1B* may be something like ethnic culture. Therefore, the high frequency of ADH1B*47His in TK and AU may have resulted from the original population differentiation, while that in SN may have resulted from the positive selection.

### Cultural or Natural Force in the Selection of ADH1B Region

The frequencies of ADH1B*47His are high in AU, TK, HM, SN, and KJ, but *ADH1B* haplotype patterns of the AU-TK group are different from those of the other three subphyla. According to Figure 5, the haplotype AGG is responsible for the high frequency of ADH1B*47His in AU-TK group, while it was haplotype AGA in HM-SN-KJ group. And from the selection analyses only AGA showed evidence of having been selected. Therefore, we conclude that the high frequency of haplotype AGG in the AU-TK group most probably did not result from positive selection, but from the random drift that occurred in their common ancestral population. The populations in AU-TK group may have maintained the high frequency of haplotype AGG of their common ancestor since they diverged.

If we check the haplotype patterns of TB and SN, two members of the Sino-Tibetan phylum, in Figure 5, some similarities between them can be found. The AGA haplotype frequency with respect to the AGG haplotype frequency is high in each population. According to the history of Han Chinese, they derived from ancient Qiang population several thousand years ago [59]. QMR (modern Qiang) may be a very old population. There are even several individuals in QMR with the ATA haplotype that would need a quite long time to appear by rare recombination events. Therefore the haplotype pattern of QMR may be most similar to that of the Sino-Tibetan ancestor. The lower haplotype frequencies of AGG and AGA in the other TB populations might result from genetic drift, and the increase in SN might result from positive selection for which we found weak evidence. The TB and SN diverged more than 5000 years ago [53]. The SN populations moved to the east and started agriculture on the plain. Both the culture and environment changed. It is not easy to determine what might have been the selective force on the Sino-Tibetan populations.

Weakly or strongly, all of the Altaic (including AT and KJ) populations show evidence of selection. The ratio of the haplotype AGA frequency with respect to AGG in Altaic is highest among the ethnic phyla. This might be a characteristic of the original Altaic population. The highest significance level for the selection significance tests appeared in three eastern populations, MVF, KOR and JPN, but we are unaware of any social or environmental similarities among these populations. KOR and JPN are agriculture populations living by the sea, while MVF is a pastoral population on the highlands. As for the other populations with evidence of positive selection, HM phylum, KUF and SKB in the Southeast Asia, the environments and cultures are even more different from the Altaic phylum. Judging from the distribution of the “selected” populations, it is clear that climate and other environmental aspects differ. Therefore it is difficult to determine how nature has influenced the allele frequency. As the distribution is ethnic-related in some extent, some cultural force of selection is understandable, though we cannot determine the common cultural factors among the populations showing the strong evidence of selection. Perhaps cultural anthropologists will shed light on this problem.

### The Selective Region and Allele

The distribution of populations showing evidence of selection at *ADH1B* is quite different from the high frequency distribution of the ADH1B*47His allele. The high significance levels for selection are only correlated with allele frequencies of the promoter SNP rs3811801, indicating that the ADH1B*47His allele has not been selected for in the absence of the derived promoter allele. Evolutionarily, the derived promoter allele at SNP 11, rs3811801, occurred on a chromosome with the ADH1B*47His allele; thus, the derived promoter allele appears together with the ADH1B*47His allele in most cases. Because the selected core haplotype AGA includes two SNPs with derived alleles, both with likely functional consequences, we cannot be sure if ADH1B*47His has been important in the positive selection on the *ADH1B* region. The core haplotypes with the promoter derived allele but without ADH1B*47His are very rare; therefore the sample is too small to test for positive selection. We can speculate that it is the combined effect of an increased activity of the enzyme caused by the ADH1B*47His allele and an increased quantity of the enzyme caused by the derived promoter allele. However, it is possible that the promoter region variant has no effect but is simply associated by chance with that chromosome that underwent selection in specific ethnic populations.

In previous case-control studies, it was found that the ADH1B*47His allele decreased the risk for alcoholism both in Asian and European populations. In the Asian populations [21]–[25] included in the alcoholism correlation studies, both derived allele frequencies of ADH1B*47His and the promoter polymorphism are high. But in the European populations [26], [27], [29], the promoter derived allele does not appear together with ADH1B*47His, implying that ADH1B*47His is solely responsible for the decrease in the risk for alcoholism. Others report that ADH1B*47His was not always related to alcoholism, especially in Taiwanese populations [32], [33], in which the frequency of the *ADH1B* promoter derived allele is very low as we revealed in our study. Some papers [28] also doubted the significance of the association between ADH1B*47His and alcoholism in European populations as the frequency of the ADH1B*47His is also low. Therefore the ADH1B*47His allele alone may not be important in changing the genetic structure of the populations.

However the decrease in the risk for alcoholism has not been argued to be the selective force, and our results argue that selection is not solely related to ADH1B*47His. The derived promoter allele may have led to the increase of the ADH enzyme expression, and then enhance the protection against some deleterious effects. The *ADH* variants are also related to some types of cancers and other serious diseases [4], [60]–[66]. Infectious disease is one of the plausible selective forces suggested by Goldman & Enoch [67]. Other diseases such as food poisoning can also have similar effects, and the populations will be susceptible if they happen to be partial to certain foods. This kind of pathology was reported in some populations in China [68]. In the case of *ADH* selection, Altaic and Hmong populations may have special food or other customs that increase the risk of a certain disease. The enhancement of the ADH enzyme caused by the ADH1B*47His may not be enough to protect the individuals from that disease, and an increased enzyme activity caused by the derived allele of the promoter variant would then be helpful. Our hypothesis is that ADH1B*47His can enhance the activity of ADH enzyme and the derived allele of the promoter variant of *ADH1B* can increase the quantity of the enzyme in the body; thus the protection against some related diseases will be stronger and the *ADH1B* haplotype will be selected. However, more case-control studies including both of the derived alleles are required. And we suggest these studies be conducted in the Daic or Austronesian populations, because there are more types of *ADH1B* core haplotypes and the linkage disequilibrium is weaker. Therefore, a false signal caused by the strong linkage disequilibrium can be avoided.

## Materials and Methods

### Populations

We collected samples of 24 populations (populations with stars behind their code in Table 1 and marked by colorful icons in Figure 1B) from six ethnic phyla. Among these populations, nine were collected from Laos, and 15 were from China. This is the first population genetics study in Laos. Laos is an inland country surrounded by all the other nations in the Peninsula. All types of populations in Peninsular Southeast Asia can be found in Laos. All individuals we collected are healthy adults without alcoholism or related disorders. Everyone signed the informed consent. Our study was approved by the Ethics Committee of the Chinese National Human Genome Center at Shanghai and the Human Investigations Committee at Yale University School of Medicine, and approved by the Laos government. The sample sizes are all large enough for meaningful frequency and haplotype estimates [69]. The population locations are also indicated in Table 1 by both county names and geographic coordinates, and also shown in the map of Figure 1B.

Table 1 also lists the information of populations previously studied [12] to give an overview of the *ADH* study in East Asia. These population samples are categorized both ethnically and geographically. Each phylum contains 2∼7 populations. HM is the smallest phylum, represented in our data by only two populations.

### Sample Preparation

For the new populations blood samples were collected from finger tips of participants and kept dry on filter paper. A whole genome amplification (WGA) [70] was applied for each sample. A 3 mm^2^ piece of paper with blood was cut off and dipped into 5 µl water, lysed with 5 µl of alkaline lysis solution (400 mM KOH, 100 mM DTT, 10 mM EDTA) and incubated for 10 min on ice. The lysed liquid was neutralized with 5 µl neutralization solution(1 M HCl∶1 M Tris-HCl = 2∶3, pH 0.6). The neutralized mixture was used directly as template in WGA. The WGA cocktail is water 31.5 µl, 10×MDA buffer [Tris-HCl pH7.5 375 mM, KCl 500 mM, MgCl_2_ 100 mM, (NH_4_)_2_SO_4_ 50 mM] 5 µl, 10 mM dNTP 5 µl, 2 mM thiophosphate-modified random hexamer (NNNN*N*N, where* denotes a phosphorothioate) 1.25 µl, 2 mg/ml Rnase A 0.25 µl, template mixture 5 µl, 200 ng/µl φ29 polymerase 2 µl. Cocktail was mixed well and incubated at 31°C for 15 hours. After the reaction, the polymerase activity was inactivated at 75°C for 5 min. The product was spun down to get rid of the sediment, and the produced DNA was purified by alcohol precipitation before TaqMan reactions.

### Markers

We chose 30 SNPs from the ADH cluster, covering *Class I ADH* (encompassing the *ADH1B* Arg47His polymorphism) and *ADH7*. These 30 SNPs have high heterozygosity in East Asia [71]. The dbSNP and ALFRED numbers of each SNP from the ADH clusters are listed in Figure 2. All SNPs were genotyped by the TaqMan method [72] using commercial assays and reagents (http://products.appliedbiosystems.com) except for the assay for *ADH1B* Arg47His, which we designed [30].

### Analysis Methods

Allele frequencies were calculated directly by gene counting assuming two-allele codominant inheritance. F_st_ values [73] are calculated as 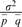. Our F_st_ values were calculated across all 32 East Asian populations. Haplotypes of the 30 SNPs in *ADH* genes were estimated by PHASE2.1 [74], [75]. The population-specific patterns of regions of high LD were calculated and graphed (Figure 2) using HAPLOT [76] and the default Kidd r^2^ partition algorithm. The PCA [77] was initially applied based on the *ADH1B* haplotype frequency of 6 SNPs (rs2066701-rs2075633-rs4147536-rs1229984-rs6810842-rs3811801) centered on *ADH1B Arg47His* by SPSS13.0. Another PCA was based on frequencies of all available haplotypes of all the 30 SNPs we typed. The second PCA was expected to show the general population relationships with less functional effects, while the first one was supposed to be affected by the functional variant. SNP allele frequency and PCA maps were presented by Surfer8.0 .

Analyses of empirical data suggest positive selection has operated on the ADH Class I cluster in East Asian populations [12] . Therefore, the Long Range Haplotype analysis [78] has been applied to test for a potential positive selection effect on these new populations. Pilot studies suggested that SNPs (9∼11: rs1229984-rs6810842-rs3811801) showed a signature of positive selection and therefore these three SNPs were selected as the core region. We used two values of Long Range Haplotype analysis to measure the selection, EHH and REHH [79].

EHH is defined as the probability that two randomly chosen chromosomes carrying a tested core haplotype are homozygous at all SNPs for the entire interval from the core region to the distance *x.* REHH is defined as the ratio of the EHH of the tested core haplotype to the EHH of the grouped set of core haplotypes at the region not including the tested core haplotype. In implementation, EHH is calculated as
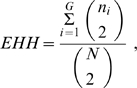
(1)where N is the total number of chromosomes/haplotype sequences, and G is the number of homozygous groups, with each group *i* having *n_i_* elements. If there are M chromosome groups, each with C_i_ chromosomes and an EHH value of EHH_i_, REHH is calculated as
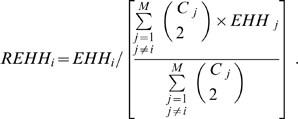
(2)


Generally speaking, if a certain core haplotype with moderate to high frequency shows high EHH and REHH over a long distance, it will be considered as an effect of positive selection [78]–[80]. The REHH value of this core haplotype was further compared with numerous data points generated by simulation that assumes neutral evolution [81]. A pilot and cursory population history mode in our simulation was designed according to the migration history (out of Africa around 50,000 years ago and arrived in East Asia around 20,000 years ago), archaeological discoveries (agriculture stared in East Asia around 8,000 ago, and iron tools were used around 2,500 years ago) and the environmental changes (the Last Ice Age ended around 12,000 years ago) in East Asia [41], [52]. The simulated data were logarithmicly transformed to achieve normality for a T-test with REHH values of our core haplotype.

The network of the core region haplotypes was drawn by NETWORK4.201 [82]. The evolutionary relationships among the haplotypes were determined from the network given the identity of the ancestral allele.

## Supporting Information

Table S1 Haplotype frequencies of the ADH region in Asian populations
(0.73 MB XLS)

Figure S1 Extended Haplotype Homozygosity (EHH) and Relative Extended Haplotype Homozygosity(REHH) of southwest populations in East Asia. Note: Colorful lines are data of core haplotype (3)AGA, and gray lines are data of other haplotypes. The data following the population codes are frequencies of the core haplotype in the populations.
(0.31 MB TIF)
