# Supplementary figures and images for: Ethnic Related Selection for an ADH Class I Variant within East Asia
Source: PLoS One. 2008 Apr 2;3(4):e1881. doi: 10.1371/journal.pone.0001881 (PMC2268739; doi:10.1371/journal.pone.0001881)

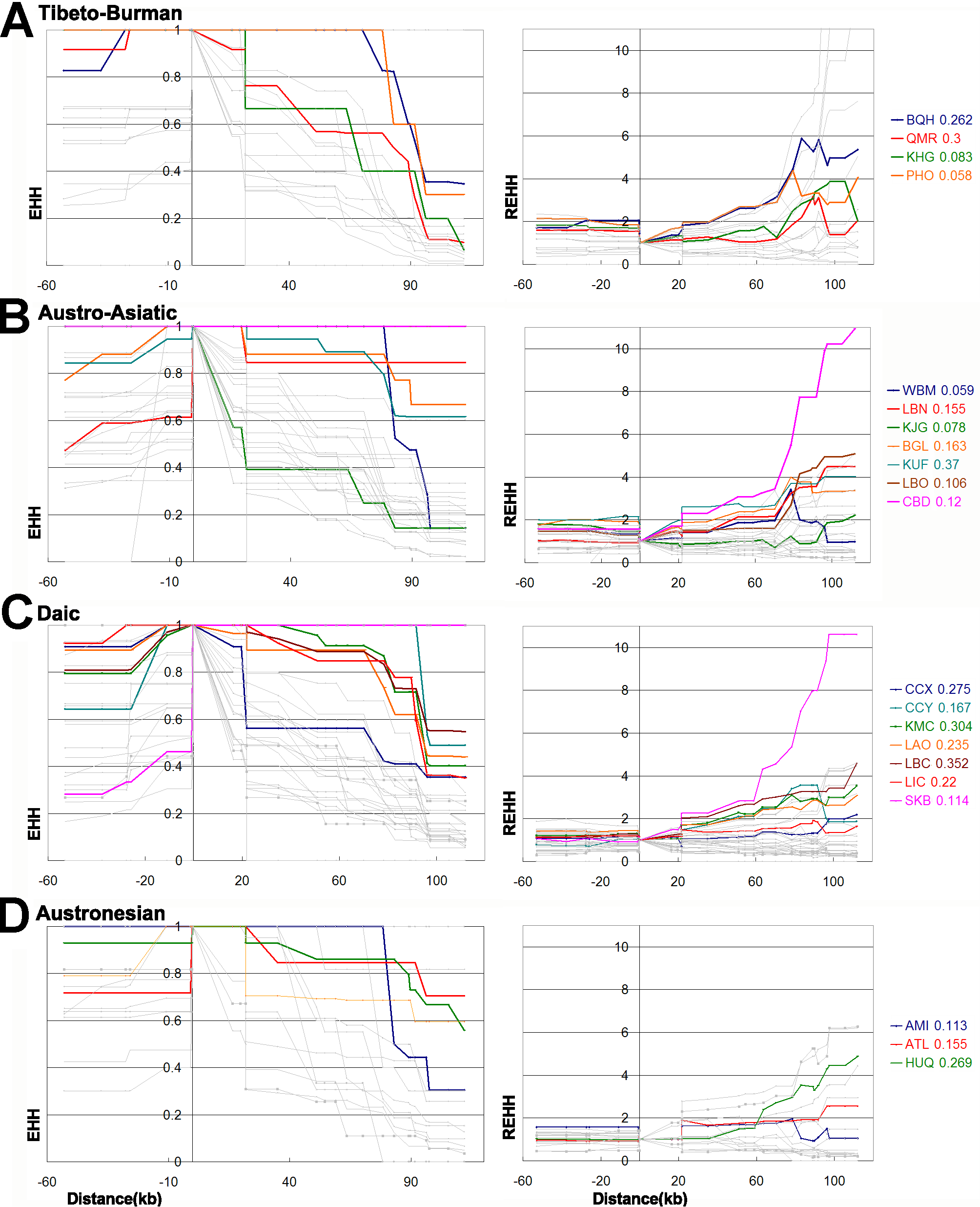

Supplement: Figure S1 — Extended Haplotype Homozygosity (EHH) and Relative Extended Haplotype Homozygosity(REHH) of southwest populations in East Asia. Note: Colorful lines are data of core haplotype (3)AGA, and gray lines are data of other haplotypes. The data following the population codes are frequencies of the core haplotype in the populations. (0.31 MB TIF) [file pone.0001881.s002.tif]
